# Nanomaterials Toward CO_2_ Reduction and Conversion

**DOI:** 10.3390/nano14201676

**Published:** 2024-10-18

**Authors:** Rafael Camarillo

**Affiliations:** Department of Chemical Engineering, Faculty of Environmental Sciences and Biochemistry, University of Castilla-La Mancha, Av. Castilla-La Mancha, s/n, 45071 Toledo, Spain; rafael.camarillo@uclm.es

## 1. Introduction

The increasing concentration of CO_2_ in the atmosphere is one of the main factors contributing to global climate change [1]. The search for effective solutions to mitigate this problem has led to research on, and the development of, innovative technologies [2]. Among these, nanomaterials have emerged as a promising tool due to their unique properties and their ability to interact at the molecular level with CO_2_ [3].

Nanomaterials, thanks to their high surface-to-volume ratio and catalytic properties, offer new avenues for the capture of CO_2_, reduction in CO_2_, and the conversion of CO_2_ into useful products [4]. These materials can be designed to have high selectivity and efficiency in the adsorption of CO_2_, as well as in its transformation into valuable chemical compounds such as methane, methanol, and other hydrocarbons [5]. Furthermore, the versatility of nanomaterials allows for their integration into various industrial systems and processes, which expands their applicability and potential impact [6].

In this Special Issue, recent advances in the use of nanomaterials for the reduction and conversion of CO_2_ are reviewed. Different types of nanomaterials are analyzed, including metal–metal oxides and carbon-based materials, highlighting their mechanisms of action and their efficiency. Likewise, current challenges and future perspectives in this field are discussed, with the aim of providing a comprehensive view of how nanomaterials can contribute to climate change mitigation through CO_2_ management.

## 2. An Overview of Published Articles

Kedruk et al. [7] studied the morphology effect of zinc oxide nanostructures on electro- and photocatalytic properties. ZnO is one of the most active semiconductors for photocatalysis and a cost-effective electrocatalyst for CO_2_ reduction. These ZnO nanostructures are synthesized by different low-cost routes (direct thermal decomposition, low-temperature chemical precipitation, and a green microwave-assisted route) and their rhodamine-B dye photocatalytic degradation and CO_2_ electrocatalytic reduction abilities are evaluated. The authors find that ZnO flakes’ morphology achieves the best results compared to nanorod or nanoparticle structures. This can be attributed to their higher aspect ratio, which gives them excellent structural, optical, and electrical properties.

Andrade et al. [8] employed a novel hydrothermal method in the presence of supercritical CO_2_ to synthesize TiO_2_ nanoparticles from different TiO_2_ precursors (diisopropoxytitanium bis(acetylacetonate), titanium (IV) isopropoxide, titanium (IV) butoxide, and titanium (IV) 2-ethylhexyloxide), and different hydrolytic agent/precursor ratios (10–40 mol/mol) using ethanol as hydrolytic agent. They conclude that the physicochemical properties of the catalysts are not significantly affected by these variables, although photocatalysts obtained from titanium (IV) isopropoxide and titanium (IV) 2-ethylhexyloxide at higher hydrolytic/precursor ratios have a higher CO_2_ photoconversion rate. These results could be due to these catalysts having an appropriate crystal size, surface area, light absorption, and charge transfer properties.

Dubadi and Jaroniec [9] describe the one-pot mechanochemical synthesis of carbons with high porosity and ordered mesopores for CO_2_ uptake at ambient conditions. This method simplifies the previous preparations and eliminates the need for toxic chemicals. Tannin and glyoxal were used as carbon precursors and two different triblock copolymers (Pluronic F127 and P123) were used as soft templating agents. Tannin to Pluronic F127 block copolymer weight ratios in the range 1:0.75–1:1.1 were used to tune the textural properties of these carbons, leading to ordered mesoporous carbons. However, Pluronic P123 leads to disordered carbon mesostructures. Some CO_2_-activated carbon samples obtained from both Pluronic templates exhibit a high specific surface area, large pore volume, narrow size distribution, and high CO_2_ uptake at ambient pressure and temperature.

Karawek et al. [10] assessed the photocatalytic conversion of CO_2_ to fuels using a TiO_2_ nanosheets/graphene oxide heterostructure as a photocatalyst. TiO_2_ nanosheets were synthesized using a hydrothermal technique in the presence of a HF soft template. These nanosheets were composited with graphene oxide and doped with copper during this hydrothermal process. The composites exhibited outstanding photoactivity when converting CO_2_ to methane and acetone. This photoactivity can be attributed to the heterostructure of the interior of the two 2D nanostructures (Cu-TiO_2_ nanosheets and graphene oxide). The interfaces of these two structures serve as n-p heterojunctions, holding active radicals for subsequent reactions. They also direct the charge transfer, promoting electron–hole separation in the photocatalyst.

Finally, Higarond et al. [11] describe the recent developments in lead and lead-free halide perovskite nanostructures towards a photocatalytic CO_2_ reduction. Perovskites exhibit extraordinary physicochemical and optical properties that make them emerging photocatalysts for CO_2_ reduction, such as a tunable bandgap, high surface energy, high charge carrier lifetime, and flexible structure. These properties can be tuned to induce crystallographic defects in perovskites. Pb halide perovskites and their composites or heterojunction with other semiconductors (metal NP, metal complexes, graphene, MOFs) have been studied in works on CO_2_ conversion. In this work, the authors review the recent progress made by Pb and Pb-free halide perovskites (to avoid the toxicity of Pb) in achieving a photocatalytic reduction in CO_2_. The influence of some important factors, such as changes in the solvent, structure defects, or composition, on the efficiency of CO_2_ conversion are also discussed.

## 3. Conclusions

This Special Issue reviews recent advances in the use of nanomaterials for the reduction and conversion of CO_2_, highlighting their potential to mitigate climate change. Nanomaterials, thanks to their high surface-to-volume ratio and catalytic properties, offer new routes for the capture and transformation of CO_2_ into useful products such as methane, methanol, and other hydrocarbons.

Different types of nanomaterials are analyzed, including metal oxides and carbon-based materials, highlighting their mechanisms of action and efficiency. For example, zinc oxide (ZnO) structures with a flake morphology can obtain better results in rhodamine-B dye photocatalysis and CO_2_ electrocatalysis due to their high aspect ratio.

Likewise, TiO_2_ nanomaterials synthesized in the presence of supercritical CO_2_ present with favorable physicochemical properties for the photoconversion of CO_2_.

Also highlighted is the synthesis of ordered mesoporous carbons using mechanochemical synthetic methods, which can simplify previous preparations and eliminate the use of toxic chemicals. These carbons show a high CO_2_ adsorption capacity under ambient conditions.

Furthermore, the photocatalytic conversion of CO_2_ to fuels is evaluated using TiO_2_/graphene oxide heterostructures, which exhibit outstanding photoactivity due to their heterogeneous structure and efficient charge separation.

Finally, recent developments in halogenated perovskites, both leaded and lead-free, are reviewed, highlighting their potential as emerging photocatalysts for CO_2_ reduction.

In summary, nanomaterials offer promising solutions for CO_2_ management, although there are still challenges and future perspectives that need to be addressed to maximize their impact on climate change mitigation.
